# Vitamin D Supplementation Ameliorates Metabolic Dysfunction in Patients with PCOS: A SystematicReview of RCTs and Insight into the Underlying Mechanism

**DOI:** 10.1155/2020/7850816

**Published:** 2020-12-19

**Authors:** Shan Guo, Reshef Tal, Haoyu Jiang, Tao Yuan, Ying Liu

**Affiliations:** ^1^Department of Reproductive Medicine, Beijing Obstetrics and Gynecology Hospital, Capital Medical University, Beijing 100026, China; ^2^Division of Reproductive Endocrinology and Infertility, Department of Obstetrics, Gynecology, and Reproductive Sciences, Yale University School of Medicine, New Haven, CT 06510, USA; ^3^Department of Neurosurgery, Beijing Tiantan Hospital, Capital Medical University, Beijing, China

## Abstract

**Objective:**

Evidence suggests that vitamin D deficiency correlated with metabolic disorders in women with polycystic ovary syndrome (PCOS). We conducted this systematic review and meta-analysis to evaluate the impact of vitamin D supplementation alone on glucose, lipid, and androgen parameters and inflammation biomarkers in women with PCOS.

**Methods:**

Literature research was conducted in Pubmed, Embase, Web of Science, Clinical Trials, and Cochrane Library to identify relevant randomized controlled trials (RCTs) up to March 2020. The effect of vitamin D supplementation alone on women with PCOS was compared with administration of placebo. The systematic review and meta-analysis protocol was registered in the International Prospective Register of Systematic Reviews (Prospero) as number CRD42020157444.

**Results:**

Thirteen randomized controlled trials with 824 patients in total were included. Serum FPG, fasting insulin, HOMA-IR, and VLDL-C were significantly decreased in the vitamin D group versus placebo. Vitamin D supplementation group also showed a significantly elevated level of QUICKI. No significant impact was seen on serum triglyceride, total-C, LDL-C, HDL-C, total testosterone, DHEAS, SHBG, or hs-CRP. Subgroup analysis demonstrated that oral vitamin D intake had significantly decreased serum triglyceride and total-C level in women with PCOS who have vitamin D deficiency (serum vitamin *D* < 20 ng/ml).

**Conclusion:**

The findings of the present meta-analysis indicate that vitamin D supplementation exerted favorable effects among women with PCOS on glucose metabolism and lipid metabolism, especially in vitamin D deficient women, but had no significant effect on the androgenic profile or inflammation status.

## 1. Introduction

Polycystic ovary syndrome (PCOS) is one of the most common endocrine disorders affecting 6–10% of reproductive age women [1]. The main clinical manifestations of PCOS are irregular menstruation, polycystic ovarian morphology, hyperandrogenism, and infertility [2]. Women with PCOS are more prone to suffer from metabolic disorders including insulin resistance, dyslipidemia, and hypertension leading to increased long-term cardiovascular risk [3]. At present, treatments for patients with PCOS include life style interventions and drug therapy aiming at restoring menstruation and providing endometrial protection, decreasing androgen levels, and lowering insulin resistance [4]. Since the etiology and pathogenesis of the syndrome remain obscure, there is no specific therapy for this population. High prevalence of PCOS and its negative influence on both physical and psychological health of women have drawn important public health concerns.

Until now, metformin has been the first-line treatment for patients with PCOS who have insulin resistance, but associated side effects such as abdominal pain, diarrhea, or headache are common [5]. It is necessary to find a safe and economical treatment for these patients. Vitamin D is responsible for bone health by enhancing intestinal absorption of calcium [6]. Its importance in reproduction has become increasingly recognized over the past decade [7]. The receptor for vitamin D is expressed in several reproductive tissues including the ovary, uterus, and placenta [8, 9]. Recent data from human studies suggests that vitamin D deficiency may be associated with reproductive disorders including PCOS. A meta-analysis comprising fourteen studies including a total of 2,262 women (1,150 patients with PCOS/1,162 controls) reported serum 25-hydroxyvitamin D was significantly lower in patients with PCOS than controls [8].

Vitamin D deficiency is defined as a 25-hydroxyvitamin (25-OH)D serum concentration of less than 20 ng/ml, while vitamin D insufficiency occurs at a level from 20 ng/ml to <30 ng/ml [10]. Numerous randomized controlled trials (RCT) have been conducted to evaluate the potential therapeutic effect of vitamin D supplementation in women with PCOS having vitamin D deficiency or insufficiency. Several published systematic review and meta-analyses have been performed, indicating that vitamin D oral supplementation had a little overall effect on metabolic status among patients with PCOS [11–16]. However, most of those articles included either non-RCTs or cosupplemented trials (with metformin or other nutrients), which may mask the real effect of vitamin D supplementation. Moreover, those published reviews tended to focus on one specific area, which may prevent us from getting a thorough understanding of the potential beneficial effects of vitamin D supplementation. Due to the conflicting results of vitamin D supplementation effects on glucose, lipid, androgenic, and inflammatory profile, we conducted a comprehensive systematic review and meta-analysis focusing on RCTs and studies only employing vitamin D supplementation alone without cosupplementation to reach a more convincing conclusion.

## 2. Methods

### 2.1. Registration

The protocol for this systematic review was registered in the International Prospective Register of Systematic Reviews (Prospero) as number CRD42020157444 [17]. This systematic review and meta-analysis process and manuscript development complied with the PRISMA guidelines [18].

### 2.2. Literature Search

Pubmed, Embase, Web of Science, Cochrane Library, and Clinical Trials were searched up to March 22, 2020, with the following MeSH and non-MeSH terms: “vitamin D”, “vitamin D3”, “vitamin D2”, “cholecalciferol”, “ergocalciferols”, “hydroxycholecalciferol” or “calcitriol” combined with “polycystic ovary syndrome”, “ovary polycystic disease”, “PCOS”, “stein-Leventhal syndrome”, “stein-Leventhal syndrome”. The reference lists of identified literatures were also browsed to identify any potential additional publications. No restrictions were made for language or date of publications.

### 2.3. Inclusion and Exclusion Criteria

Inclusion criteria were as follows: (1) RCTs; (2) study subjects were women diagnosed with PCOS; (3) PCOS was diagnosed on the basis of the 2003 Rotterdam criteria or the 1990 National Institute of Child Health and Human Development criteria; (4) full text was accessible; (5) studies comparing the therapeutic effect of vitamin D supplement with placebo.

Exclusion criteria were as follows: (1) studies examining the effects of vitamin D combination with other interventions such as metformin, calcium, oral contraceptive, and so on; (2) studies examining the effects of vitamin D among patients with PCOS undergoing intrauterine insemination (IUI) or in vitro fertilization (IVF) treatment; (3) incomplete data; (4) genetic research.

### 2.4. Study Selection and Data Extraction

Literature searches were conducted by two reviewers (S. G. and H. Y. J.) separately, and then the title and abstract were screened for eligibility. Full texts retrieved were carefully checked according to the inclusion and exclusion criteria to select qualified trials independently. Disputes were solved through discussion with another reviewer (T. Y.), and consensus was reached.

Data extraction were carried out by two reviewers (S. G. and H. Y. J.), and the following information was extracted: the last name of the first author, publishing year, country, criteria used for diagnosis of PCOS, study population, sample size, type and duration of intervention, dose of vitamin D intake, serum vitamin D level, biochemical indices of glucose and lipid metabolism including fasting plasma glucose (FPG), fasting insulin, HOMA-IR, QUICKI, serum triglyceride (TG), total cholesterol (TC), high-density lipoprotein cholesterol (HDL-C), very low-density lipoprotein cholesterol (VLDL-C), hypersensitive C-reactive protein (hs-CRP), total testosterone (Total T), sex hormone-binding globulin (SHBG), and dehydroepiandrosterone sulfate (DHEAS). Any discrepancies were resolved by discussion with another reviewer (T. Y.). In the event of incomplete information, authors were contacted to acquire relevant data.

### 2.5. Quality Assessment

The Cochrane risk of bias assessment tool was employed to evaluate the method of randomization, allocation of concealment, blinding of participants, personnel and outcome assessment, incomplete outcome, selective reporting, and other biases. Risk bias of each study was graded as low, unclear, and high. Any dispute was resolved by consensus.

### 2.6. Statistical Analysis

Statistical synthesis and subgroup analysis were performed by Revman V.5.3 offered by the Cochrane Collaboration. Measurement data were displayed as mean difference and standard deviation. Mean difference (MD) with 95% confidence intervals (CI) by inverse variance method was employed in case of data with identical measuring units; otherwise, standard mean difference (SMD) was adopted. Heterogeneity among studies was estimated by Cochran's Q test and I-squared. *P* < 0.10 with *I*^2^ > 50% indicates statistical heterogeneity. When significant heterogeneity presented, the random-effects model of meta-analysis was applied. Publication bias was assessed by funnel plots.

## 3. Results

### 3.1. Literature Search

**Figure 1:**
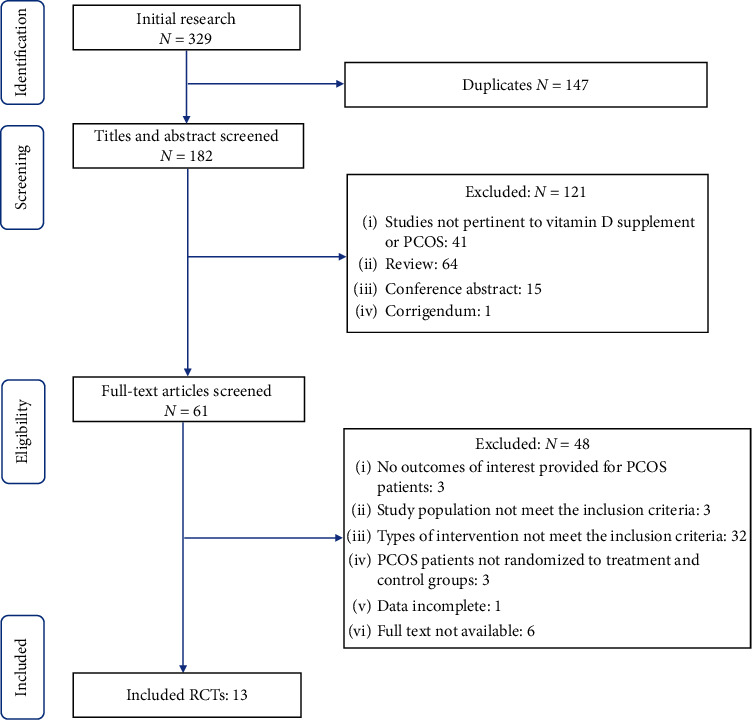
Flowchart of search strategy.

The online database search yielded 329 articles. After removing 147 duplicates, 182 articles were excluded by screening the title and abstract, and then, the remaining 61 articles were inspected carefully for eligibility. Studies without outcomes of interest (*n* = 3), study population (*n* = 3), and types of intervention (*n* = 32: concomitant metformin use, *n* = 5; concomitant OCP use, *n* = 1; concomitant other nutrients use, *n* = 26) not accordant with the inclusion criteria, studies without control group (*n* = 3), studies with incomplete data (*n* = 1), and studies without full text (*n* = 6) were excluded. Finally, 13 RCTs were selected according to the inclusion and exclusion criteria. The flowchart detailing selection of studies is presented in Figure 1.

### 3.2. Characteristics of Included RCTs

**Figure 2:**
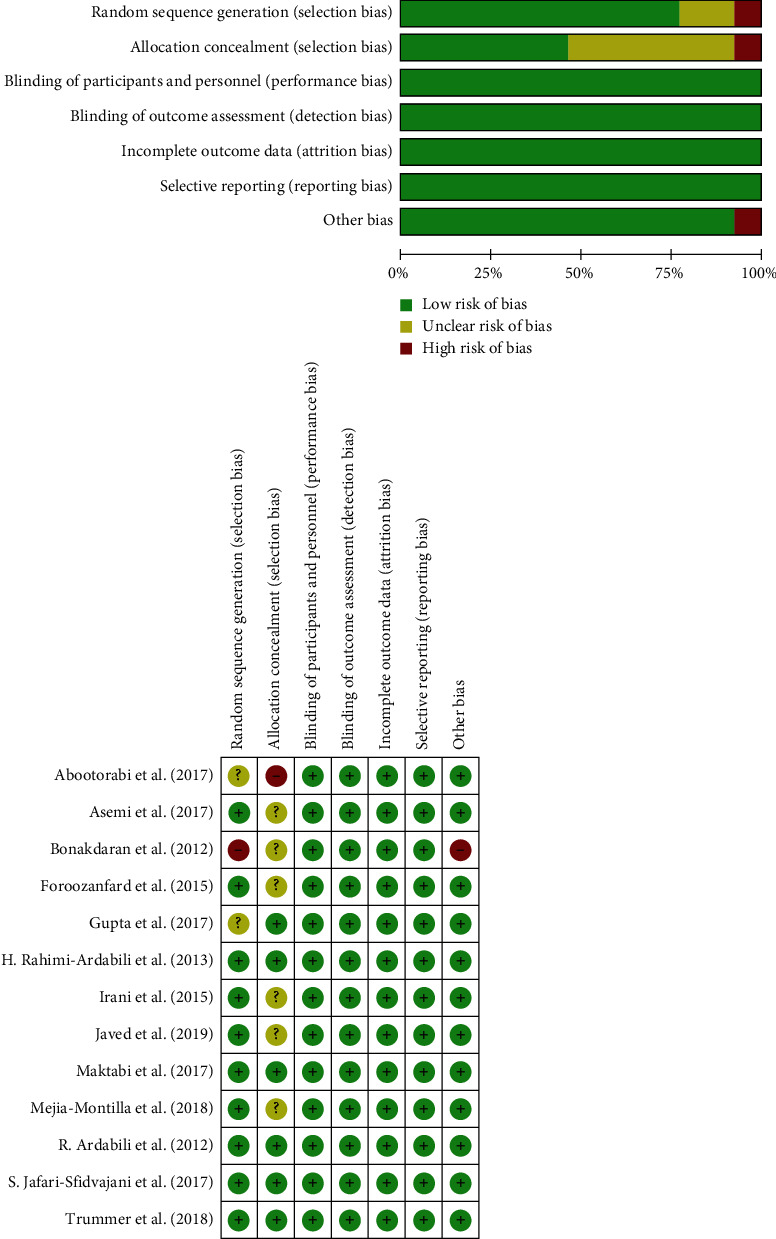
Quality assessment for randomized controlled trials included on basis of Cochrane risk of bias assessment tool.

These papers were published from 2012 to 2019 and conducted in Iran [19–26], India [27], Austria [28], Venezuela [29], the United Kingdom [30], and the United States [31] A total of 824 women with PCOS aged 18–40 years were included, and the duration of intervention was 8 weeks, 12 weeks, or 24 weeks. The dose of vitamin D intake varied from 2000 IU/week to 50,000 IU/week. Essential features of trials included in the meta-analysis are summarized in Table 1. The overall risk of included trials was estimated to be low. The quality evaluation of included trials is shown in Figure 2.

### 3.3. Serum Vitamin D Levels

**Figure 3:**
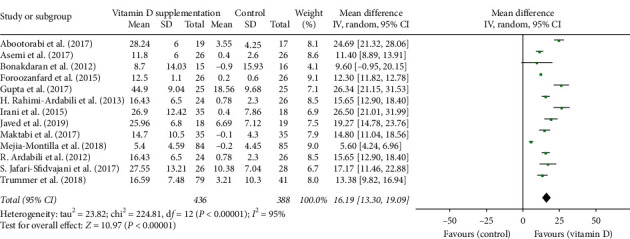
Forest plot of vitamin D concentration in experimental and placebo groups.

Twelve studies reported significant increase in serum vitamin D level following supplementation. The baseline serum level of 25(OH)D in intervention group ranged from 3.5 ± 4.2 ng/ml to 19.55 ± 6.73 ng/ml, indicating that selected patients with PCOS were mostly vitamin D deficient. Combination of data extracted from thirteen studies revealed a significant increase in 25(OH)D concentrations in the vitamin D treatment group versus placebo (MD: 16.19 ng/ml, 95% CI: 13.30, 19.09) (Figure 3).

### 3.4. Glucose Metabolism Biomarkers

**Figure 4:**
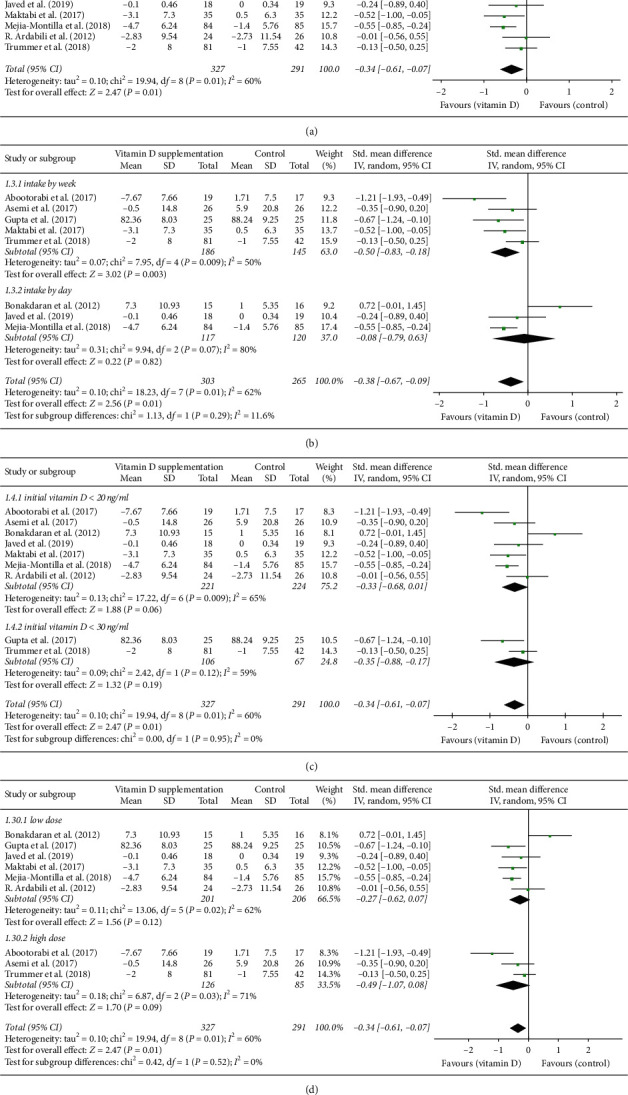
(a) Forest plot of FPG level in experimental and placebo groups. (b) Forest plot of FPG level in women with PCOS supplemented by day, week, and 20 days. (c) Forest plot of FPG level in women with PCOS who have vitamin D deficiency or insufficiency. (d) Forest plot of FPG level in women with PCOS supplemented with low- or high-dose vitamin D.

Nine studies were evaluated for FPG level. Meta-analysis showed serum FPG level of patients with PCOS significantly decreased after vitamin D supplementation (SMD: −0.34, 95% CI: −0.61, −0.07) (Figure 4(a)). Heterogeneity between studies was high (*P*=0.01, *I*^2^=60%). The form of vitamin D agent was one of the sources of heterogeneity, and the heterogeneity could be eliminated after removal of the trial adding calcitriol (Bonakdaran et al.) [26]. The beneficial effect on FPG level became more evident taking intake manner into account (intake by week, SMD: −0.50, 95% CI: −0.83, −0.18) (Figure 4(b)) and was independent of the extent of baseline vitamin D deficiency among patients with PCOS (vitamin *D* < 20 ng/ml, SMD: −0.33, 95% CI: −0.68, 0.01; vitamin *D* < 30 ng/ml, SMD: −0.35, 95% CI: −0.88, 0.17) (Figure 4(c)) and supplementation dose (low dose, SMD: −0.27, 95% CI: −0.62,0.07; high dose, SMD: −0.49, 95% CI: −1.07, 0.08) (Figure 4(d)).

**Figure 5:**
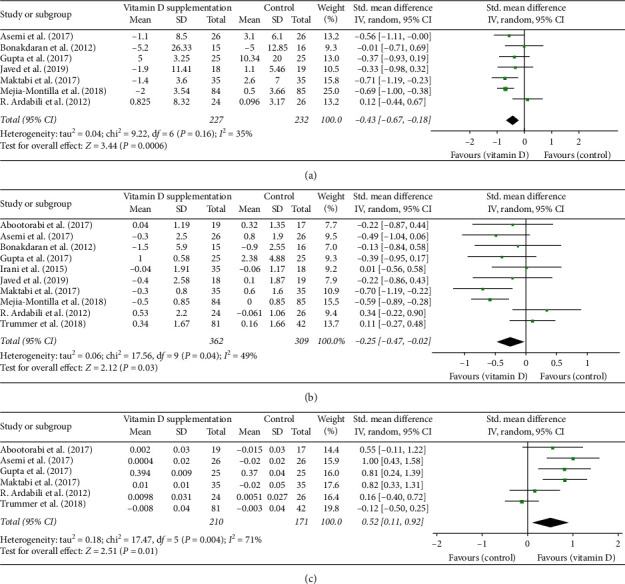
(a) Forest plot of fasting insulin level in experimental and placebo groups. (b) Forest plot of HOMA-IR in experimental and placebo groups. (c) Forest plot of QUICKI in experimental and placebo groups.

In total, ten studies have assessed insulin resistance indices in women with PCOS, among which seven studies reported concentration of fasting insulin, and ten studies reported HOMA-IR and six studies evaluated QUICKI. We found a significant decrease in serum fasting insulin level (SMD: −0.43, 95% CI: −0.67, −0.18) (Figure 5(a)) and HOMA-IR (SMD: −0.25, 95% CI: −0.47, −0.02) (Figure 5(b)). Meanwhile, a significant increase in QUICKI was also observed (SMD: 0.52, 95% CI: 0.11, 0.92) (Figure 5(c)). To further evaluate the potential influence of extent of vitamin D deficiency at baseline and intake interval on the observed effects, subgroup analyses were carried out and revealed that vitamin D supplementation was effective in ameliorating insulin resistance in women with PCOS who have baseline serum vitamin *D* < 20 ng/ml (fasting insulin, SMD: −0.40, 95% CI: −0.69, −0.10; Figure S1(a)) (HOMA-IR, SMD: −0.25, 95% CI: −0.47, −0.02; Figure S1(b)) (QUICKI, SMD: 0.52, 95% CI: 0.11, 0.92; Figure S1(c)). In addition, the effect was related to intake interval. Daily vitamin D oral intake was found to be efficacious in lowering HOMA-IR (SMD: −0.46, 95% CI: −0.72, −0.20; Figure S1(d)) and fasting insulin level (SMD: −0.44, 95% CI: −0.85, −0.04; Figure S1(e)). Meanwhile, weekly supplementation also resulted in a significant decrease in fasting insulin level (SMD: −0.56, 95% CI: −0.87, −0.26; Figure S1(e)) but had no effect on QUICKI (SMD: −0.53, 95% CI: −0.04, 1.11; Figure S1(f)). When supplementation dose was taken into consideration, low dose supplementation (<50,000 IU/week) improved fasting insulin level (SMD: −0.39, 95%CI: −0.68, −0.11; Figure S1(g)), QUICKI (SMD: 0.61, 95%CI: 0.18, 1.03; Figure S1(h)), and HOMA-IR (SMD: −0.33, 95%CI: −0.63, −0.02; Figure S1(i)) in patients with PCOS. The heterogeneity within studies was low in selected indices except for QUICKI (*P*=0.004, *I*^2^=71.0%). When study population was stratified by serum vitamin D concentration, the heterogeneity became low.

### 3.5. Lipid Metabolism Biomarkers

**Figure 6:**
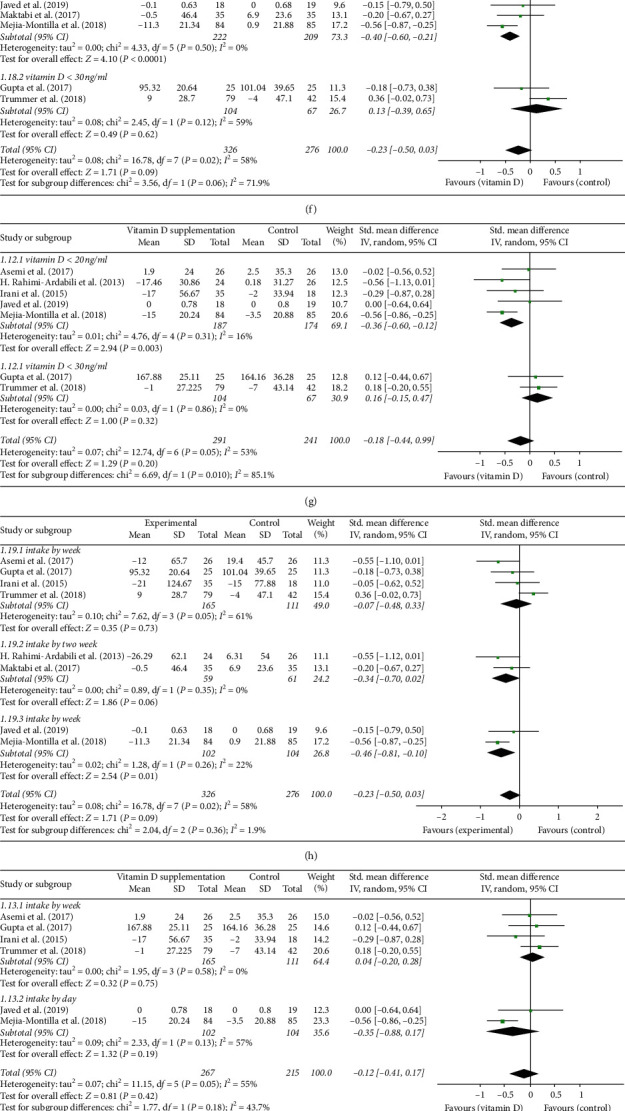
(a) Forest plot of total-C level in experimental and placebo groups. (b) Forest plot of LDL-C level in experimental and placebo groups. (c) Forest plot of VLDL-C level in experimental and placebo groups. (d) Forest plot of HDL-C level in experimental and placebo groups. (e) Forest plot of triglycerides in experimental and placebo groups. (f) Forest Plot of triglycerides in women with PCOS who have vitamin D deficiency or insufficiency. (g) Forest plot of total-C in women with PCOS who have vitamin D deficiency or insufficiency. (h) Forest plot of triglycerides in women PCOS supplemented by day, week, two weeks, and 20 days. (i) Forest plot of total-C in women with PCOS supplemented by day, week, and 20 days. (j) Forest plot of triglycerides in women with PCOS supplemented with a low or high dose. (k) Forest plot of total-C in women with PCOS supplemented with a low dose or high dose. (l) Forest plot of LDL-C in women with PCOS supplemented with a low dose or high dose.

A total of eight trials were included for assessing the effect of vitamin D supplementation on lipids, all of which reported serum triglyceride levels. Among these studies, seven trials reported serum concentrations of total-C and HDL-C, six trials reported results for LDL-C, and three trials examined serum level of VLDL-C. The overall effect of vitamin D supplementation significantly lowered serum VLDL-C level (MD: −3.83 mg/dl, 95% CI: −7.34, 0.32; Figure 6(c)) but did not affect serum concentration of total-C (SMD: −0.18, 95% CI: −0.44, 0.09; Figure 6(a)), LDL-C (SMD: −0.23, 95% CI: −0.60,0.14; Figure 6(b)), HDL-C (SMD: 0.15, 95% CI: −0.03, 0.33; Figure 6(d)), or triglycerides (SMD: −0.23, 95% CI: −0.50, 0.03; Figure 6(e)). We also examined whether study population, intake interval, and supplementation dose were involved in vitamin D supplementation influence on lipid metabolic parameters. We found evidence that vitamin D supplementation reduced triglycerides (SMD: −0.40, 95% CI: −0.60, −0.21; Figure 6(f)) and total-C (SMD: −0.36, 95% CI: −0.60, −0.12; Figure 6(g)) significantly in vitamin D deficient patient group. Dosing interval was associated with the decrease of serum triglycerides (intake by day, SMD: −0.46, 95% CI: −0.81, −0.10; Figure 6(h)) but had no effect on total-C level (intake by week, SMD: 0.04, 95% CI: −0.20, 0.28; intake by day, SMD: −0.35, 95% CI: −0.88, 0.17; Figure 6(i)). Similarly, both low dose of vitamin D supplementation (<50,000 IU/week) or high dose (>50,000 IU/week) were not associated with significant changes in triglycerides (low dose, SMD: −0.21, 95% CI: −0.54, 0.12; high dose, SMD: −0.30, 95% CI: −0.79, 0.18; Figure 6(j)), total-C (low dose, SMD: −0.18, 95%CI: −0.54, 0.18; high dose, SMD:−0.15, 95% CI: −0.54, 0.24; Figure 6(k)), or LDL-C (low dose, SMD: −0.37, 95%CI: −0.80, 0.06; high dose, SMD: 0.06, 95%CI: −0.33, 0.46; Figure 6(l)) level. There was heterogeneity among studies when data on triglycerides (*P*=0.02, *I*^2^=58%), total-C (*P*=0.05, *I*^2^=53%), and LDL-C (*P*=0.005, *I*^2^=70%) were combined. Subgroup analysis found that heterogeneity resulted from discrepancy in vitamin D level of patients with PCOS and sample size.

### 3.6. Androgenic and Inflammatory Profile

**Figure 7:**
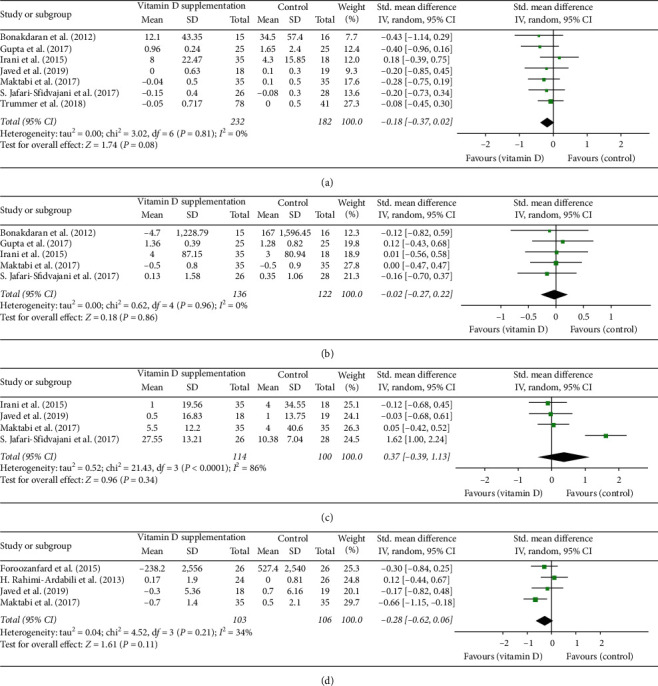
(a) Forest plot of total testosterone level in experimental and placebo groups. (b) Forest plot of DHEAS level in experimental and placebo groups. (c) Forest plot of SHBG level in experimental and placebo groups. (d) Forest plot of hs-CRP level in experimental and placebo groups.

Seven studies in total reported on serum androgens, of which five studies had results for total testosterone concentration, five trials had data on DHEAS, and four trials examined serum SHBG level. The results of the meta-analysis revealed that vitamin D supplementation in women with PCOS did not have significant effect on total testosterone (SMD: −0.18, 95% CI: −0.37, 0.02; Figure 7(a)), DHEAS (SMD: −0.02, 95% CI: −0.27, 0.22; Figure 7(b)), and SHBG (SMD: 0.37, 95% CI: −0.39, 1.13; Figure 7(c)). In addition, four studies evaluated effects on hs-CRP, and the results of our meta-analysis showed that vitamin D supplementation decreased serum concentration of hs-CRP significantly (SMD: −0.28, 95%CI: −0.62, 0.06; Figure 7(d)).

### 3.7. Publication Bias

**Figure 8:**
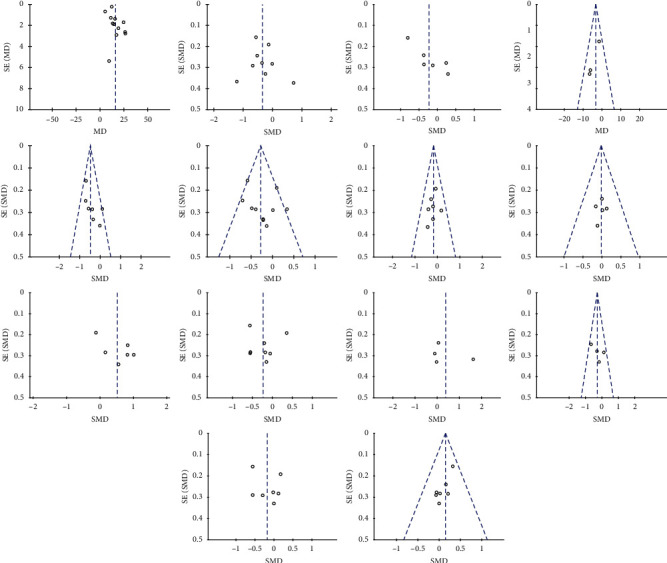
Funnel plot of standard error by standard differences in the means of plasma: (a) vitamin D; (b) FPG; (c) fasting insulin; (d) HOMA-IR; (e) QUICKI; (f) triglycerides; (g) total-C; (h) HDL-C; (i) LDL-C; (j) VLDL-C; (k) total testosterone; (l) DHEAS; (m) SHBG; (n) hs-CRP in selected trials.

The funnel plots for serum vitamin D concentrations indicated a risk for lack of reporting on negative effect of vitamin D supplementation. The funnel plots for the rest of the indices showed there was no significant publication bias. However, for each result, the number of studies of meta-analysis was less than 10, which may be too small to determine publication bias through funnel plots (Figures 8(a)–8(n).

## 4. Discussion

Several meta-analyses have been conducted concerning the effect of vitamin D supplementation on metabolic biomarkers of women with PCOS, suggesting variable beneficial effects, but the results remained conflicting. Moreover, the data of non-RCTs and cosupplementation trials were combined in most of these reviews. In view of the increasing number of RCTs regarding this topic, we conducted the present meta-analysis of RCTs focusing on the effect of vitamin D intake alone without cosupplementation compared with placebo to reach more convincing conclusions. The results of our meta-analysis revealed that vitamin D oral intake alone improved insulin resistance parameters and reduced inflammation in patients with PCOS. Furthermore, subgroup analysis showed that lipid metabolism was also improved in vitamin D deficient group. No effects were found on serum androgen levels or inflammation status.

Vitamin D deficiency is very prevalent in women with PCOS. A recent cross-sectional study demonstrated that, compared with fertile controls, significantly lower vitamin D levels were present in women with PCOS (mean 25(OH)D of 64.5 nmol/l vs. 49.0 nmol/l, resp.); meanwhile, higher HOMA-IR and lipid abnormalities are associated with deficient vitamin D levels [32]. The recommended dose of vitamin D supplementation by the Endocrine Society Guidelines is 50,000 IU once weekly for eight weeks [33]. In our meta-analysis, most of the included patients with PCOS had insufficient vitamin D level (<30 ng/ml). After vitamin D supplementation, serum 25(OH)D concentrations in patients with PCOS increased significantly with a mean difference of 16.19 ng/ml (95% CI: 13.30, 19.09). The heterogeneity of this response is high (*I*^*2*^ = 95%, *P* < 0.00).

Evidence from our meta-analysis showed that vitamin D supplementation resulted in lowering blood fasting glucose levels in addition to improving insulin resistance, as seen by a significant decrease in serum fasting insulin and HOMA-IR along with a slight increase in QUICKI. The results of our meta-analysis are in contrast with earlier ones by other researchers. Xue et al. [11] and Fang et al. [12] failed to find a positive effect of vitamin D supplementation on glucose metabolism in women with PCOS. Additionally, in a more recent meta-analysis, vitamin D supplementation was found to affect glucose and HOMA-IR significantly only when cosupplemented with other nutrients [16]. Several reasons may be underlying these differences. First, the meta-analysis by Lagowska et al. incorporated RCT studies that included diverse means of intervention with different kinds of micronutrients in addition to vitamin D, which may explain more variable treatment effects [16]. Second, prior meta-analyses did not exclude studies in which commonly used medications to treat PCOS such as metformin and OCPs were used [12, 13, 16]. The use of such medications known to ameliorate insulin resistance in the placebo control group may diminish the observed effects of vitamin D intervention. Third, prior meta-analyses have also included a number of single-arm before–after studies [11], which could make the pooled results less robust. To further study the optimum way of oral vitamin D administration, we conducted subgroup analysis revealing that daily intake could reduce serum glucose and insulin level, while low-dose intake could alleviate insulin resistance conditions. The potential mechanism of the effect of vitamin D on glucose metabolism may be the result of both genomic and nongenomic effects. Increased25(OH)D enhances VDR signaling helping pancreatic *β*-cells restore function through BRD7/PBAF pathway [34]. The 1, 25(OH)2D-VDR complex binds to the vitamin D response element of the insulin receptor in tissue improving insulin responsiveness for glucose transport and suppressing the release of proinflammatory cytokines, which are believed to mediate IR [34]. Moreover, its influence on the extracellular and intracellular calcium regulation is vital for the modulation of glucose transport in target tissues [35].

Notably, decline in serum triglycerides, total-C, and VLDL-C concentration were significant among patients with PCOS who are deficient in vitamin D in our meta-analysis. This finding is in accordance with the significant improvement of serum triglycerides level observed in the meta-analysis by Xue et al. [11]. However, in Xue's article, this effect was observed with low dose vitamin D supplementation (<50,000 IU/week) rather than high dose. However, in our study, subgroup analysis showed that a low dose of vitamin D supplementation had no impact on triglycerides or total-C level, which means that high-dose vitamin D intake may be more beneficial for lipid metabolism. A clarified mechanism that can explain the beneficial effects of vitamin D on lipid metabolism is that increased intracellular calcium in the liver leads to the stimulation of microsomal triglyceride transfer protein, which participates in the formation and secretion of VLDL, resulting in decreased circulating levels of serum total-C [25]. Additionally, in a randomized controlled trial, it was shown that serum VEGF levels and TGF-*β*1 to soluble endoglin (sENG) ratio is significantly decreased by vitamin D supplementation in women with PCOS [31, 36]. The decrease in VEGF and TGF-*β*1 : sENG ratio correlated with the decrease in triglycerides in the PCOS group. Increased circulating TGF-*β*1 levels have been associated with various cardiometabolic conditions including obesity [37], diabetes [38], and coronary artery disease [39]. It has been suggested that higher levels of insulin, LH, and androgens increase the concentration of VEGF, leading to abnormally increased vascularity in the ovary, which may exacerbate anovulation and subfertility. TGF-*β*1 upregulates the secretion of VEGF, and the resulting angiogenic imbalance may play a role in the development of metabolic syndrome and cardiovascular disease in women with PCOS [40].

In this meta-analysis, we did not find any improvement in serum total testosterone, DHEAS, or SHBG level. Similar findings were reported in the meta-analysis by Azadi-Yazdi et al. [14]. In their study, significant decrease in serum testosterone concentration was only found in single-arm before– after studies, but not in high-quality RCTs. Meanwhile, in the current meta-analysis, we found that serum hs-CRP level did not change significantly after vitamin D supplementation. Previously, a meta-analysis conducted by Akbari et al. [15] reported that vitamin D supplementation resulted in an improvement in hs-CRP of women with PCOS, but in this article, the significant decrease was evident after including three trials examining the anti-inflammatory effect of vitamin D and other nutrition. Our meta-analysis demonstrated that vitamin D supplementation alone did not influence hs-CRP level among women with PCOS.

**Figure 9:**
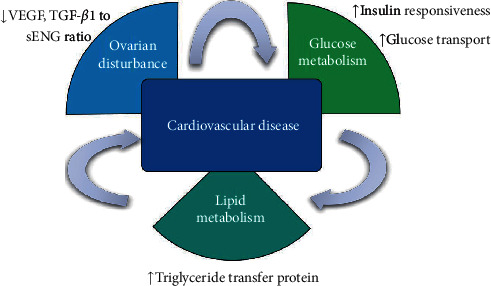
Potential mechanism of therapeutic effect of vitamin D on women with PCOS.

Our meta-analysis has several strengths. It includes comprehensive research of RCTs and strict inclusion of high-quality studies, in which vitamin D intervention was given alone. This allowed for a more focused analysis of vitamin D effects in PCOS. Our work also has some limitations. There is considerable heterogeneity between studies with variable race/ethnicity and age. In addition, baseline vitamin D status, vitamin D dose, and formulation and duration of treatment of vitamin D vary between studies and are an additional source of confounding. Moreover, most of the trials included were conducted in Iran, where women are covered by clothes resulting in less exposure to sunlight, so whether the benefits of vitamin D supplementation can be generalizable to women with PCOS from all over the world is still an open question. In addition, considering the limited number of high-quality RCTs and small sample size, the conclusions of the present meta-analysis should be extrapolated with caution. However, as an inexpensive and safe treatment option, vitamin D supplementation can be implemented in women with PCOS who have vitamin D deficiency. Our study may shed light on the potential mechanisms behind those discovered benefits that vitamin D deficiency may be a codeterminant factor in metabolism disorder. Taken together, vitamin D supplementation appears to influence several aspects of clinical features present in women with PCOS (Figure 9).

## 5. Conclusion

Our systematic review showed that oral intake of vitamin D supplementation attenuated insulin resistance and hyperlipemia, but not androgenic profile or inflammatory markers in women with PCOS who have vitamin D deficiency. Several potential mechanisms may be underlying these beneficial effects of vitamin D on clinical features of PCOS, and further research is needed to explore the complex role of vitamin D in different pathways of this metabolic disorder.

## Figures and Tables

**Table 1:** Summary of RCTs focusing on the effect of Vitamin D supplementation in women with polycystic ovary syndrome.

Author/year/country	Population/age/vitamin D level	Intervention	Duration	25OHD before treatment(g/ml)	25OHD after treatment (ng/ml)	Hyperandrogenism	Insulin resistance	Dyslipidemia	Inflammation
Seyyed et al., (2017) Iran [19]	36 women with PCOS, aged 20–38 years, 25OHD < 20 ng/ml	G1: 50,000 IU/week of oral vitamin D3 (*n* = 19); G2: placebo (*n* = 17)	8 weeks	G1: 3.5 ± 4.2; G2: 9.8 ± 5.1	28.24 ± 6.47; 13.3 ± 7.1		↓FPG ↔HOMA-IR, QUICKI		

Asemi et al., (2017) Iran [21]	104 overweight and obese women with PCOS, aged 18–40 years, 25OHD < 20 ng/ml	G1: 1000 mg/d calcium + vitamin D placebo (*n* = 26); G2: 50,000 IU/week vitamin D + calcium placebo (*n* = 26); G3: 1000 mg/d calcium+ 50,000 IU/week vitamin D (*n* = 26); G4: calcium placebo + vitamin D placebo (*n* = 26)	8 weeks	G1: 13.9 ± 2.0; G2: 11.6 ± 4.7; G3: 15.1 ± 3.6; G4: 14.0 ± 4.1	71.2 ± 14.7; 86.8 ± 16.1; 76.4 ± 13.3; 73.5 ± 23.8		↓insulin, HOMA-IR, ↑QUICKI ↔FPG	↓TG, VLDL-C ↔TC, LDL-C, HDL-C	8 weeks

Bonakdaran et al., (2012) Iran [26]	48 women with PCOS, aged 20–40 years, 25OHD < 20 ng/ml	G1: 1000 mg/d metformin (*n* = 17); G2: 0.5ug/d calcitriol (*n* = 15); G3: placebo (*n* = 16)	12 weeks	G1:28.2 ± 13.5; G2:11.4 ± 8.2; G3:19.9 ± 16.5;	26.7 ± 10.6; 20.1 ± 16.2; 19.0 ± 15.3	↔Total testosterone, DHEAS	↔FPG, insulin, HOMA-IR		

Foroozanfard et al., (2015) Iran [22]	104 overweight women with PCOS who have vitamin D deficiency, aged 18–40 years, 25OHD < 20 ng/ml	G1: 1000 mg calcium/d plus vitamin D placebo weekly (*n* = 26); G2: 50,000 IU/week vitamin D plus calcium placebo daily (*n* = 26); G3: 1000 mg/d calcium plus 50,000 IU vitamin D weekly (*n* = 26); G4: calcium placebo daily plus vitamin D placebo weekly (*n* = 26)	8 weeks		G1: +0.3 ± 0.4; G2: +12.5 ± 1.1; G3: +9.2 ± 1.5; G4: +0.2 ± 0.6				↓hs-CRP

Gupta et al., (2017) India [27]	50 women with PCOS, aged 18–45 years, 25OHD < 30 ng/ml	G1: 12000 IU/week vitamin D (*n* = 25); G2: placebo (*n* = 25)	12 weeks	G1: 18.56 ± 9.68; G2: Vacant	44.90 ± 9.04; vacant	↔-Total testosterone, DHEAS	↓FPG, insulin, HOMA-IR ↑ QUICKI	↔TG, TC, HDL-C	

Rahimi-Ardabili et al., (2013) Iran [25]	50 women with PCOS, aged 20–40 years, 25OHD < 20 ng/ml	G1: 50,000 IU/20 days of oral cholecalciferol (*n* = 24); G2: placebo (*n* = 26)	8 weeks	G1:6.9 ± 2.8; G2:7.28 ± 2.93	23.4 ± 6.14; 8.57 ± 3.98			↓ TC ↔TG, LDL-C, HDL-C, VLDL-C	↓hs-CRP
Javed et al. (2019) UK [30]	37 women with PCOS, aged 18–45 years, 25OHD < 20 ng/ml	G1: 3200IU/day vitamin D (*n* = 18); G2: placebo (*n* = 19)	12 weeks	G1:10.26 ± 4.57; G2:12.38 ± 4.45	36.22 ± 7.81; 19.07 ± 8.21	↔Total testosterone, SHBG	↔FPG, insulin, HOMA-IR	↔TG, TC, LDL-C, HDL-C	↔ hs-CRP

Irani et al., (2015) USA [31]	53 women with PCOS, aged 18–38 years, 25OHD <20 ng/ml	G1: 50,000 IU/week of oral vitamin D3 (*n* = 35); G2: placebo (*n* = 18)	8 weeks	G1: 16.3 ± 0.9; G2: 17 ± 1.8	43.2 ± 2.4; 17.4 ± 1.9	↔Total testosterone, SHBG	↔ HOMA-IR	↓TG ↔HDL-C, LDL-C	

Maktabi et al., (2017) Iran [24]	60 women with PCOS, aged 18–40 years, 25OHD < 20 ng/ml	G1: 50,000 IU/2 weeks of oral vitamin D3 (*n* = 30); G2: placebo (*n* = 30)	12 weeks	G1: 12.8 ± 4.5; G2: 14.5 ± 5.1	27.5 ± 9.8; 14.4 ± 5.2	↔Total testosterone, SHBG, DHEAS	↓FPG, insulin, HOMA-IR, QUICKI	↔TG, TC, LDL-C, HDL-C, VLDL-C	↓hs-CRP

Jorly Mejia-Montilla et al., (2018) Venezuela [29]	169 women with PCOS, aged 20–40 years, 25OHD < 20 ng/ml	G1: 5000 IU/day vitamin D (*n* = 84); G2: placebo (*n* = 85)	12 weeks	G1:13.7 ± 4.2; G2:13.5 ± 4.4	19.1 ± 4.9; 13.3 ± 4.5		↓FPG, insulin, HOMA-IR	↓TG, TC, LDL-C ↔HDL-C	

Ardabili et al., (2012) Iran [20]	50 women with PCOS, aged 20–40 years, 25OHD < 20 ng/ml	G1:50,000 IU of oral vitamin D3 every 20 days (*n* = 24); G2: placebo (*n* = 26)	8 weeks	G1: 6.9 ± 2.8; G2: vacant	23.4 ± 6.1; vacant		↔FPG, insulin, HOMA-IR, QUICKI		

Jafari-Sfidvajani et al., (2017) Iran [23]	56 women with PCOS, aged 20–40 years, 25OHD < 20 ng/ml	G1: low-calorie diet+ 50,000 IU/week oral vitamin D3 (*n* = 26); G2: low-calorie diet + placebo (*n* = 28)	12 weeks	G1: 15.83 ± 4.85; G2: 14.93 ± 5.55	43.38 ± 12.61; 25.32 ± 10.13	↔Total testosterone, DHEAS, SHBG			

Trummer et al., (2018) Austria [28]	123 women with PCOS, aged ≥ 18 years, 25OHD < 30 ng/ml	G1:20,000 IU of oral vitamin D3/week (*n* = 81); G2: placebo (*n* = 42)	24 weeks	G1: 19.55 ± 6.73; G2: 19.55 ± 7.01	36.14 ± 8.05; 22.76 ± 11.82	↔Total testosterone	↔ FPG, HOMA-IR, QUICKI	↔TG, TC	

IU: international units; FPG: fasting plasma glucose; IR: insulin resistance; HOMA-IR: homeostasis model of assessment-estimated insulin resistance; QUICKI: quantitative insulin sensitivity check index; HDL-C: high-density lipoprotein cholesterol; LDL-C: low-density lipoprotein cholesterol; VLDL-C: very low-density lipoprotein cholesterol; TC: total cholesterol; TG: triglyceride; hs-CRP: high sensitive C-reactive protein; SHBG: sex hormone-binding globulin; DHEAS: dehydroepiandrosterone sulfate.

## Data Availability

The data used in the study are available upon request to the corresponding author.

## Supplementary Materials

Supplementary Materials Supplementary Figure S1: (a) forest plot of fasting insulin level in women with PCOS who have vitamin D deficiency or insufficiency; (b) forest plot of HOMA-IR in women with PCOS who have vitamin D deficiency or insufficiency; (c) forest plot of QUICKI in women with PCOS who have vitamin D deficiency or insufficiency; (d) forest plot of HOMA-IR in women with PCOS supplemented by day, week, two weeks, and 20 days; (e) forest plot of fasting insulin in women with PCOS supplemented by day, week, two weeks, and 20 days; (f) forest plot of QUICKI in women with PCOS supplemented by week, two weeks, and 20 days; (g) forest plot of fasting insulin level in women with PCOS supplemented with low- or high-dose vitamin D; (h) forest Plot of QUICKI in women with PCOS supplemented with low- or high-dose vitamin D; (i) forest plot of HOMA-IR in women with PCOS supplemented with low- or high-dose vitamin D.
