# Supplementary material for: Vitamin D Supplementation Ameliorates Metabolic Dysfunction in Patients with PCOS: A SystematicReview of RCTs and Insight into the Underlying Mechanism
Source: Int J Endocrinol. 2020 Dec 19;2020:7850816. doi: 10.1155/2020/7850816 (PMC7773476; doi:10.1155/2020/7850816)
Supplement: Supplementary Materials — Supplementary Figure S1: (a) forest plot of fasting insulin level in women with PCOS who have vitamin D deficiency or insufficiency; (b) forest plot of HOMA-IR in women with PCOS who have vitamin D deficiency or insufficiency; (c) forest plot of QUICKI in women with PCOS who have vitamin D deficiency or insufficiency; (d) forest plot of HOMA-IR in women with PCOS supplemented by day, week, two weeks, and 20 days; (e) forest plot of fasting insulin in women with PCOS supplemented by day, week, two weeks, and 20 days; (f) forest plot of QUICKI in women with PCOS supplemented by week, two weeks, and 20 days; (g) forest plot of fasting insulin level in women with PCOS supplemented with low- or high-dose vitamin D; (h) forest Plot of QUICKI in women with PCOS supplemented with low- or high-dose vitamin D; (i) forest plot of HOMA-IR in women with PCOS supplemented with low- or high-dose vitamin D. [file 7850816.f1.docx]

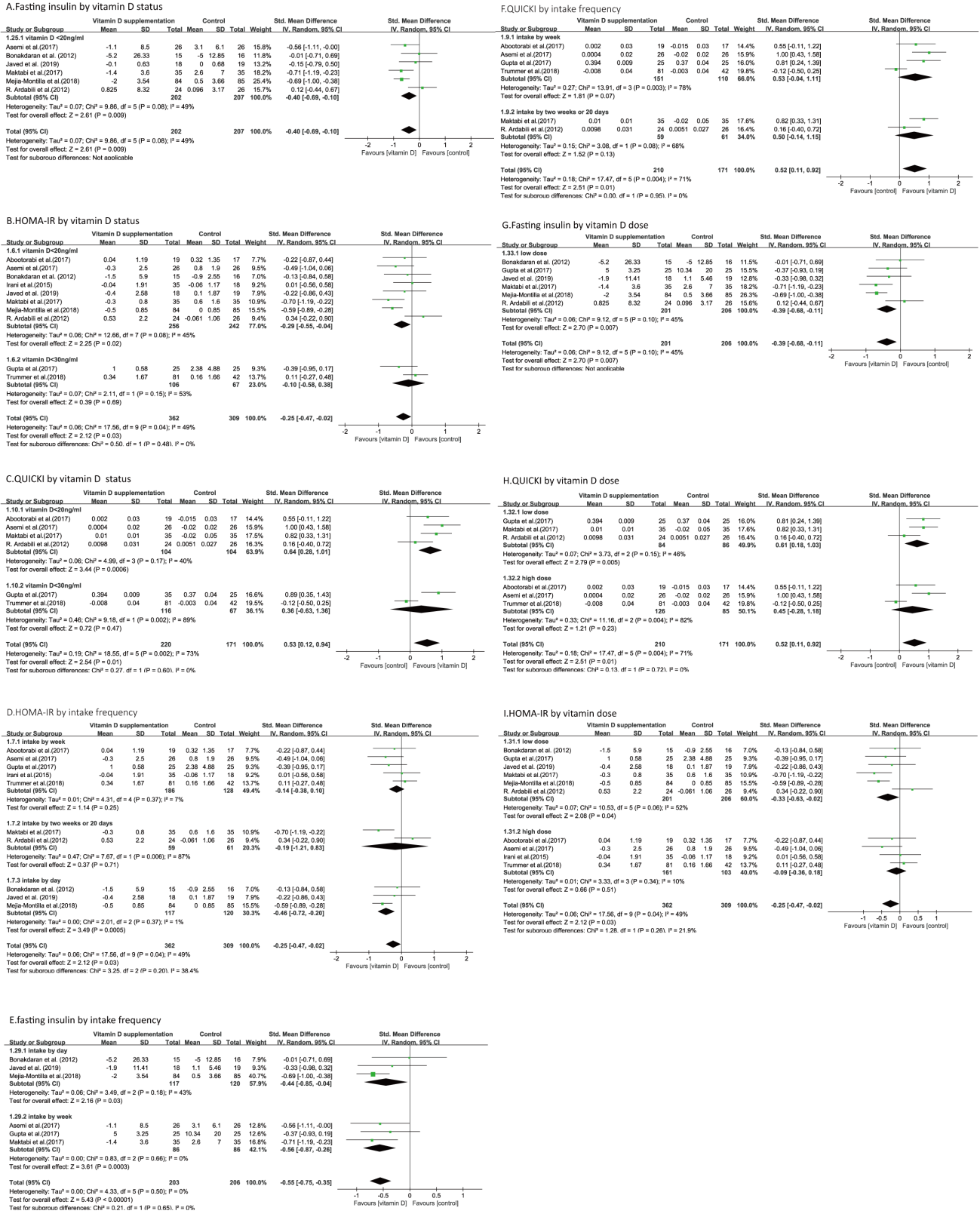


Supplementary figure S1 (A) Forest Plot of fasting insulin level in PCOS women with vitamin D deficiency or insufficiency. (B) Forest Plot of HOMA-IR in PCOS women with vitamin D deficiency or insufficiency. (C) Forest Plot of QUICKI in PCOS women with vitamin D deficiency or insufficiency. (D) Forest Plot of HOMA-IR in PCOS women supplemented by day, week, two weeks, 20days. (E) Forest Plot of fasting insulin in PCOS women supplemented by day, week, two weeks, 20days. (F) Forest Plot of QUICKI in PCOS women supplemented by week, two weeks, 20days. (G) Forest Plot of fasting insulin level in PCOS women supplemented with low or high dose vitamin D. (H) Forest Plot of QUICKI in PCOS women supplemented with low or high dose vitamin D. (I) Forest Plot of HOMA-IR in PCOS women supplemented with low or high dose vitamin D.
